# P-1907. Barriers to Prescribing Long-Acting Injectable HIV Pre-Exposure Prophylaxis in a Resident Primary Care Clinic

**DOI:** 10.1093/ofid/ofaf695.2076

**Published:** 2026-01-11

**Authors:** Michael Z Chen, Aniruddha Hazra

**Affiliations:** University of Chicago, Chicago, IL; University of Chicago, Chicago, IL

## Abstract

**Background:**

HIV pre-exposure prophylaxis (PrEP) is up to 99% effective at reducing the risk of acquiring HIV, now available in oral and long-acting injectable (LAI) forms. Uptake of PrEP, especially LAI PrEP, varies among patient groups and is lower among Black patients, particularly Black men who have sex with men. At the University of Chicago Medical Center (UCMC) resident primary care clinic, internal medicine (IM) residents act as primary care physicians for patients who are majority Black and LAI PrEP is under-prescribed in this clinic. This study aims to identify knowledge deficits in and barriers to prescribing LAI PrEP by IM residents.

**Figure:**
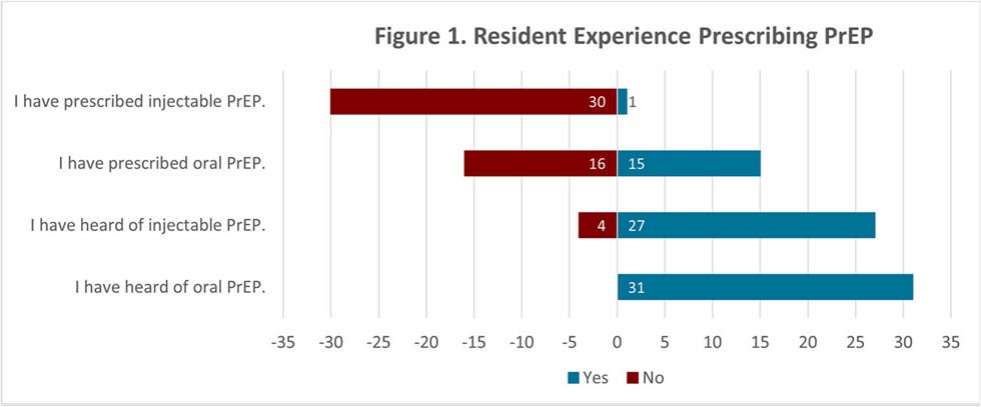

**Figure:**
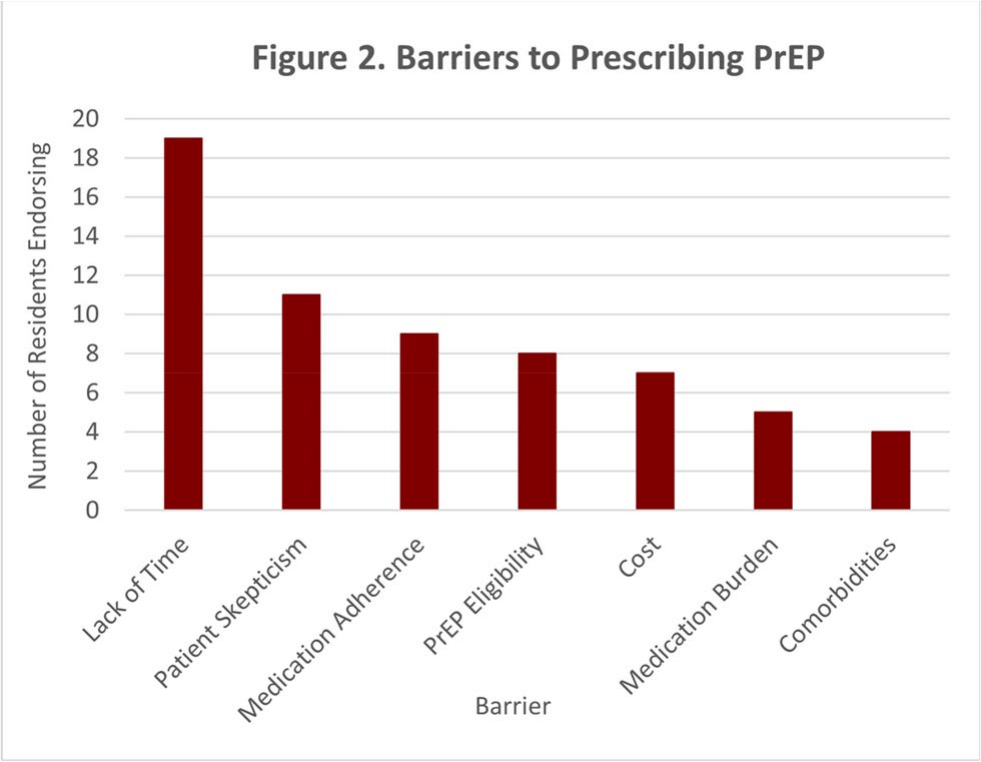

**Methods:**

IM residents at UCMC at any training level (n=118) were surveyed for experiences prescribing oral and injectable PrEP for primary care patients. Experience prescribing PrEP was surveyed using yes/no questions. Attitudes towards and barriers to prescribing PrEP were surveyed using Likert scale questions. Knowledge of eligibility criteria for starting PrEP and of routine monitoring labs for continuing PrEP were assessed using true/false/unsure questions. Differences in knowledge were compared using χ^2^ tests.

**Figure:**
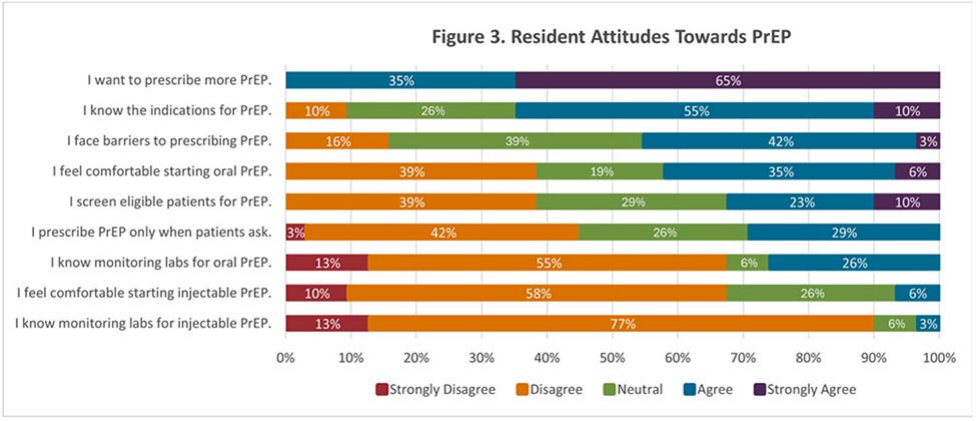

**Figure:**
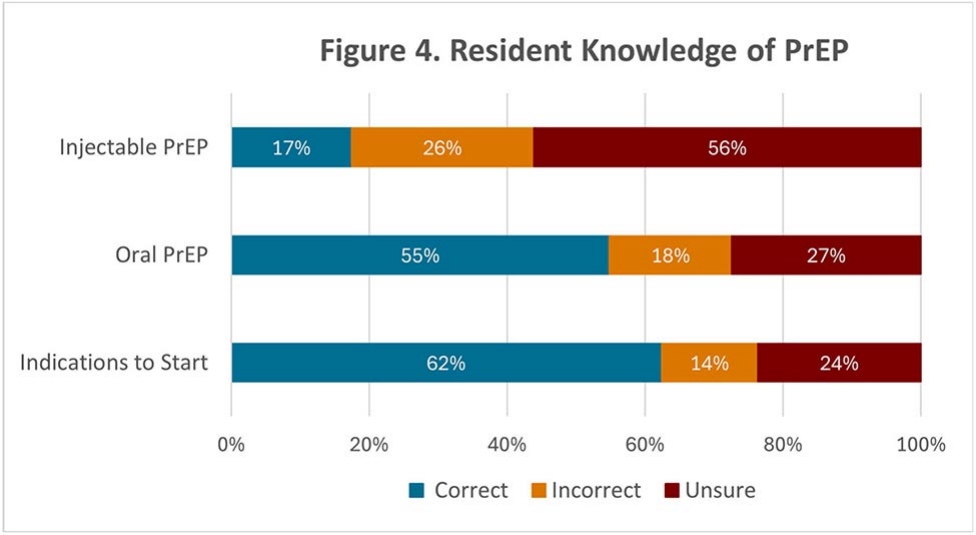

**Results:**

Of the IM residents, 26% completed the survey (n=31). Though 48% of these residents had previously prescribed oral PrEP, only 3% had prescribed LAI PrEP and 13% were unaware that LAI PrEP was available (Figure 1). 100% of residents expressed interest in prescribing PrEP more often (Figure 1). Residents cited lack of time, patient skepticism, and medication adherence as common barriers to prescribing LAI PrEP (Figure 2). Residents reported greater confidence in managing and understanding oral PrEP than LAI PrEP (Figure 3). The knowledge assessment revealed a significant difference in understanding between oral and LAI PrEP with residents answering 55% of oral PrEP questions correctly versus 17% for LAI PrEP (*p*< 0.01, Figure 4).

**Conclusion:**

IM residents demonstrated high confidence in and moderate knowledge about oral PrEP, but low confidence in and low knowledge about injectable PrEP. These results can guide development of educational materials targeting knowledge deficits in management of LAI PrEP. Such educational interventions may improve uptake of LAI PrEP in vulnerable patients in the resident primary care clinic setting.

**Disclosures:**

Aniruddha Hazra, MD, Gilead Sciences: Advisor/Consultant|Gilead Sciences: Grant/Research Support|ViiV Healthcare: Advisor/Consultant

